# Plain Packaging: One of the Plain Simple Solutions for Nicotine Reduction in the Middle East

**DOI:** 10.3390/ijerph23070879

**Published:** 2026-07-06

**Authors:** Mohammed Al-Hamdani, Steven M. Smith, Rana Abouzoor, Courtney McKay

**Affiliations:** 1 Department of Public Health, College of Health Science, QU Health, Qatar University, Doha 2713, Qatar; malhamdani@qu.edu.qa (M.A.-H.); rana.abouzoor@qu.edu.qa (R.A.); 2 Department of Psychology, Saint Mary’s University, 923 Robie Street, Halifax, NS B3H 3C3, Canada; courtney.mckay@smu.ca

**Keywords:** plain packaging, tobacco labels, tobacco packaging, health policy, cigarette packaging

## Abstract

**Highlights:**

**Public health relevance—How does this work relate to a public health issue?**

- Tobacco use remains prevalent across the Middle East, representing a prevailing preventable cause of morbidity and mortality despite existing control measures.
- A limited number of countries (Saudi Arabia and Turkey) have implemented plain packaging policies, while others (e.g., Iran) are considering adoption, highlighting a regional gap and missed opportunity to strengthen tobacco control.

**Public health significance—Why is this work of significance to public health?**

- Plain packaging is an evidence-based, low-cost intervention that reduces the appeal of tobacco products, enhances warning salience, and supports cessation efforts, making it highly suitable for implementation.
- The underutilization of plain packaging in the Middle East presents an untapped potential to accelerate declines in tobacco use and complement existing policies.

**Public health implications—What are the key implications or messages for practitioners, policy makers and/or researchers in public health?**

- Scaling up plain packaging across the region could serve as a practical and cost-effective policy that strengthens current tobacco control strategies and addresses challenges posed by emerging tobacco products.
- Adoption of plain packaging may serve as a momentum builder toward more comprehensive “endgame” strategies aimed at substantially reducing tobacco use to near-negligible levels.

**Abstract:**

Tobacco use remains a major public health challenge and continues to be highly prevalent across the Middle East despite the implementation of conventional tobacco-control policies, including taxation, smoking restrictions, advertising bans, and graphic warning labels. Although these measures have contributed to reductions in tobacco use, additional strategies are needed to further reduce prevalence rates. This manuscript presents a narrative review examining plain packaging as a tobacco-control policy option for Middle Eastern countries. Plain packaging standardizes tobacco product packaging through the use of uniform fonts and colors on a plain background and is recommended by the World Health Organization Framework Convention on Tobacco Control. This narrative review synthesizes existing literature and regional policy developments to outline six key reasons supporting the adoption of plain packaging in the Middle East: evidence on effectiveness, underutilization, cost-free implementation, its role as a starting point toward tobacco endgame strategies, public support, and challenges posed by emerging nicotine products. Overall, plain packaging represents a viable and underutilized policy option that could strengthen existing tobacco-control efforts and support long-term reductions in tobacco use across the Middle East.

## 1. Introduction

Tobacco use continues to pose serious challenges to public health [1]. Despite these challenges, tobacco use remains widespread in the Middle East, and several policy opportunities are missing [2]. The fundamental policies of taxation, banning smoking in public places, and advertisement restrictions are routine and effective in other regions [3]. Other fundamental policies, include graphic warning labels, employing a vivid image accompanied with a text warning, have shown some effectiveness in different studies relative to simple text-based messaging [4]. Graphic warnings are commonly used on tobacco products [5]. The larger the size of the warning the more effective it is in terms of getting visual attention [6], and eliciting an unfavorable physiological response [7]. Although these policies have contributed to reductions in tobacco use, they remain insufficient on their own. There is a growing global body of scientific evidence indicating that plain packaging is an effective tobacco control measure; however, the majority of syntheses and evaluations have been conducted in high-income Western settings. These settings differ greatly from the Middle East in terms of policy implementation and enforcement. The Middle East has its own unique tobacco control situation, with different rules, varying levels of how strictly they enforce the FCTC, a strong tobacco industry presence, and social and cultural rules that may affect how people accept and respond to packaging changes. Only a few countries in the Middle East and North Africa have adopted this policy. These countries include Saudi Arabia, Turkey (2019), Isreal (2020) [8,9,10], and Oman (2024) [8]. While Syria is scheduled to implement the policy in 2027 [8]. Additional countries, such as Iran [11], have expressed consideration for adopting plain packaging; however, these discussions have thus far remained largely unactioned.

The World Health Organization [WHO] obliges the members of the Framework Convention on Tobacco Control (FCTC), which includes all Middle Eastern countries, to recommend plain packaging in addition to graphic warnings [12]. Plain packaging refers to the standardization of packages such that they are displayed in standard font and color on a mono-colored background. This approach has been demonstrated to be an effective way to reduce the prevalence of tobacco use [13]. Plain packaging is a viable policy option for tobacco control [13], and is considered a tobacco “endgame” policy. Tobacco endgame policies are those that take tobacco control steps further towards a world that is tobacco-free [14]. Despite WHO’s encouragement and the growing number of countries adopting plain packaging worldwide, implementation in the Middle East remains limited. Adoption varies considerably among FCTC member states, with political, financial, and sociocultural factors strongly influencing the translation of WHO guidelines into national policy. As stated earlier, only limited number of countries in the region, including Saudi Arabia, Turkey, Isreal, and Oman [8,9,10], have instituted standardized packaging policies, yet the implementation of these policies varies in scope and enforcement. This regional variation underscores a pivotal dearth of evidence concerning the perception, implementation, and challenges of plain packaging policies within Middle Eastern contexts. Attempts to introduce plain packaging in the region have sometimes faced resistance. For example, in 2019, when plain packaging was implemented in Saudi Arabia, smokers started a social media campaign condemning the decision and claimed that the new packs contained cigarettes that did not taste as good [15]. In light of these evident gaps and regional diversities, the objective of this narrative review is to synthesize extant evidence and policy developments concerning plain packaging in the Middle East. This review aims to address the following: first, to clarify the effectiveness of plain packaging; second, to identify the challenges to implementation; and third, to highlight opportunities for reinforcing tobacco control strategies.

### Plain Packaging of Tobacco Products and the Ottawa Charter for Health Promotion

Developed by the World Health Organization (WHO) in 1986, the Ottawa Charter for Health Promotion remains one of the most widely recognized and influential frameworks in public health [16]. The WHO defines health promotion as the process of empowering individuals to enhance their autonomy and well-being. The Ottawa Charter outlines five key areas of action: developing healthy public policies, fostering supportive communities, promoting community engagement, cultivating personal skills, and restructuring health services [16]. Of these, the clearest connection between plain packaging and the Charter is the development of healthy public policy. The Charter underscores the importance of prioritizing health as a primary concern for policymakers across all sectors and underscores the potential of legislation to safeguard public health [16]. Plain packaging constitutes a compelling illustration of this strategy, as governments legislate the regulations that govern the presentation of tobacco products to consumers. The policy prioritizes health over commercial interests by not allowing tobacco companies to use packaging as a marketing tool. In 2012, Australia became the first country to implement plain packaging for tobacco products, a policy that has since been adopted by countries such as the United Kingdom, France, New Zealand, and Canada [17]. Evidence from these countries shows that plain packaging reduces product appeal, makes health warnings more noticeable, and strengthens smokers’ intentions to quit [17]. Graphic health warnings and plain packaging operate as complementary tobacco control measures. While plain packaging removes promotional branding and reduces the appeal of tobacco products, graphic warnings communicate the health risks through vivid imagery and text [17]. Their combined effect is particularly powerful: standardized packaging enhances the visibility and impact of graphic warnings, while the warnings reinforce the deterrent function of plain packaging [17]. Together, these measures create a synergistic strategy that both diminishes the marketing power of tobacco packaging and strengthens public awareness of its harm [17].

Plain packaging also aligns with the Ottawa Charter’s objective of fostering supportive environments. The environments in which people live, work, and socialize influence health. Tobacco product packaging can present smoking as attractive, appealing, or socially acceptable, particularly to adolescents and young adults, and the emerging market of young women in the Middle East [18]. Historically, tobacco packaging has used color, design, and descriptors to create associations with lifestyles and identities that target young people and reinforce the normalization of smoking [19,20]. The supportive environments action of the Ottawa Charter provides a guiding framework for policies such as plain packaging, which transform tobacco packaging from a marketing tool into a deterrent and constant reminder against tobacco use.

Additionally, plain packaging has the capacity to strengthen community action when implemented alongside public health campaigns, advocacy initiatives, and tobacco control programs led by civil society organizations. This aligns directly with the third action area of the Ottawa Charter, strengthening community action, which emphasizes empowering communities to take collective responsibility for health promotion. In several Middle Eastern countries, particularly Saudi Arabia and across the Gulf region, tobacco control efforts have been reinforced through collaborations among government agencies, healthcare institutions, educational sectors, and community groups. Within this context, plain packaging operates as a visible policy instrument that communities can rally around, mobilizing support for tobacco control and youth protection. For instance, Hassounah et al. (2020) [15] documented that following the introduction of plain packaging in Saudi Arabia, social media discussions intensified and attracted widespread engagement from consumers, tobacco companies, health advocates, media outlets, and government agencies such as the Saudi Food and Drug Authority and the Ministry of Commerce. This case underscores the critical importance of effective public communication and active stakeholder involvement in ensuring the successful implementation of tobacco control policies [15].

Plain packaging contributes to the Ottawa Charter’s fourth action area, developing personal skills, by improving access to health information and enabling individuals to make more informed choices about tobacco use. The removal of distracting branding and the enhanced visibility of graphic health warnings allow risks to be communicated more effectively. Evidence from Saudi Arabia showed that the ability to read and understand health warnings was significantly associated with smokers’ intentions to quit, while plain packaging combined with graphic warning images was widely perceived as motivating cessation among a substantial proportion of smokers [21]. Another Turkish study showed that stronger graphic health warnings and plain packaging both elicited significantly greater negative emotional and avoidant responses compared with older branded packs [10]. While these measures enhanced attention to health risks, the analysis revealed that neither plain packaging nor new warnings alone had a distinct effect on smokers’ intentions to quit within six months [10]. Standardized packaging has been found to reduce appeal, create more realistic perceptions of harm, and reduced people’s positive association [22]. These findings support the goal of tobacco control to reduce the influence of marketing. Reducing positive association has shown to weaken the normalization of smoking among young people; similarly graphic health warnings and plain packaging have increased adolescent awareness of the dangers of tobacco use and decreased cigarette pack appeal [23,24]. Messages with graphic health warnings were seen as more effective than text-only messages, especially those showing severe health problems like lung cancer and oral disease [23]. Strong emotional responses, including fear, anxiety, shock, and guilt, are evoked by these warnings, thus contributing to reduce tobacco initiation among non-smokers, and motivating current smokers quit [23].

Finally, plain packaging supports the reorientation of health services by complementing cessation and prevention strategies within healthcare systems and public health programs. By increasing the visibility and impact of health warnings, standardized packaging reinforces cessation messages delivered through clinical practice and community health initiatives. Evidence from Saudi Arabia demonstrated that plain packaging combined with health warning messages was associated with stronger intentions to quit smoking, indicating its potential to strengthen broader cessation and prevention efforts [21]. When integrated into comprehensive tobacco control programs, plain packaging contributes to shifting health services away from treatment-focused approaches toward prevention and health promotion, in line with the Ottawa Charter’s fifth action area of reorienting health services. Thus, plain packaging serves as a comprehensive tobacco control tool that not only deters the initiation and continuation of smoking but also strengthens awareness of tobacco-related harms, supports informed decision-making, and empowers individuals to adopt healthier behaviors, contributing across all five Ottawa Charter action areas by building healthy public policy, creating supportive environments, strengthening community action, developing personal skills, and reorienting health services toward prevention and health promotion.

## 2. Methods

This study was conducted as a narrative review, aiming to synthesize representative evidence on the effectiveness of plain packaging in tobacco control. Although not systematic, transparent and reproducible procedures were followed to enhance credibility.

Databases searched: We searched PubMed, Scopus, Web of Science, ProQuest, and Embase, supplemented by regional sources such as the Eastern Mediterranean Index Medicus (IMEMR). Grey literature was identified through WHO reports, government publications, and advocacy organization documents (e.g., Framework Convention Alliance, Cancer Council Victoria).

Search terms and timeframes: The search strategy combined keywords related to plain packaging, tobacco control, graphic health warnings, policy implementation, and behavioral impacts, alongside the names of Middle Eastern countries (Bahrain, Iran, Iraq, Israel, Jordan, Kuwait, Lebanon, Oman, Qatar, Saudi Arabia, Syria, the United Arab Emirates, Yemen, Egypt, Turkey, Cyprus, and Palestine). Terms were applied across all databases without language restrictions. The timeframe covered publications from 2010 to 2026, reflecting the period during which plain packaging policies were introduced and evaluated globally.

Inclusion and exclusion criteria: We included studies that directly examined plain packaging policies and their impacts on tobacco use, perceptions, or policy effectiveness. Both peer-reviewed articles and authoritative reports were eligible. Studies focusing exclusively on other tobacco control measures (e.g., taxation, advertising bans) without reference to plain packaging were excluded.

Study selection process: Representative studies that particularly mention plain packaging, especially, in the Middle East or examples of studies outside the Middle East were selected to illustrate key themes rather than exhaustively summarize all available literature. Priority was given to high-quality evaluations from countries that have implemented plain packaging (e.g., Australia, Ireland, Saudi Arabia, Turkey, Hong Kong), as well as systematic reviews and WHO reports. These examples were chosen to provide a balanced picture of behavioral impacts, packaging perceptions, and policy effectiveness across diverse contexts.

## 3. Results

We present the results in synthesized form and organize them under six key reasons for presenting the case for plain packaging policies, and why they might be particularly effective across Middle Eastern countries.

### 3.1. Evidence on Effectiveness of Plain Packaging

Empirical studies have found that plain packaging reduces the willingness to pay for tobacco products, increases the salience of package warnings, and amplifies the use of quitting helplines following implementation [25,26]. Evidence from Australia, Saudi Arabia, and the UK have shown that plain packaging featuring dark colours and standardized fonts reduces users’ enjoyment of cigarettes, contributes to declines in smoking prevalence and tobacco consumption, and discourages smoking initiation among non-smokers and occasional users [15,26,27,28,29].

The success of plain packaging has been noted across different nicotine products. For example, it has been used on cigarette packaging, e-cigarettes, and other nicotine-based products [30]. Tobacco control policymakers and researchers can look to the success found in plain-packaging studies on other substances to inform policy decisions moving forward. Evidence shows that the level of plain packaging matters—the plainer the package, in terms of stronger elimination of brand imagery and stringent standardization of fonts and colors, the more effective it is in enhancing health warning recognition and increasing negative product perceptions [31,32]. Studies examining the effectiveness of plain packaging depict higher negative perceptions, attention to warnings, support for legislation, quit attempts and quit call volume—clearly supporting this policy measure (as summarized in [33]). The use of plain packaging on e-cigarettes is associated with reduced interest in trying the products in young populations [34], as well as reduced product appeal [35]. Furthermore, studies on snus (a moist, smokeless tobacco product that is held in the mouth, either between the lips and teeth or cheek and teeth) packaging have shown that standardizing the packaging with health warnings can influence how appealing the product is, brand differentiation, and customer perception [36]. Regulatory efforts are being made to reinforce warning labels on snus and improve communication of health risks in the European Union [37]. A qualitative study in Egypt found that plain packaging coupled with larger graphic warnings are perceived to stall initiation and encourage quit attempts for waterpipe smoking [38].

Plain packaging has proven effective even with distally related addiction products as well [39]. Despite the level of social acceptability towards alcohol, studies conducted on plain packaging of alcohol products found that it led to decreased appeal and increased intention to quit [39]. Moreover, it reduced alcohol consumption and increased understanding of health risks and fear arousal [39]. Further warnings and plain packaging have also been proposed for cannabis products [40].

Altogether, the evidence demonstrates the benefits of graphic health warnings and the standardized colours, font, and shape of plaining package could be instrumental for the effectiveness for tobacco control efforts. To contextualize the discussion within the Middle East, Table 1 provides selected examples of key studies on plain packaging and tobacco warning labels conducted across the region. These studies span Saudi Arabia, Qatar, Jordan, and Turkey, among others, and employ diverse methodologies including cross-sectional surveys, qualitative case studies, randomized experiments, and policy reviews. Collectively, they highlight both the effectiveness of plain packaging in reducing product appeal and enhancing warning salience, as well as the challenges of consumer resistance, cultural perceptions, and industry interference. The evidence underscores the importance of strengthening tobacco control policies in the Middle East through standardized packaging and more impactful health warnings.

### 3.2. Underutilization

Following Australia’s introduction of plain packaging in 2012, other countries such as the United Kingdom, France, and Ireland have adapted similar laws into legislation [8]. However, most countries in the Middle East still do not have plain packaging policies, except for Saudi Arabia [8], Turkey, Isreal [12], and Oman [49]. Implementing plain package laws was a challenging but significant effort in both Saudi Arabia and Oman. For Oman, the effort to enact these laws was a several years long fight, which included on-going dynamic conversation between regional, national, and global stakeholders [49]. In Saudi Arabia the high prevalence of tobacco use in both youth and adult male categories increased the need for anti-smoking measures, which included plain packaging requirements to be enacted [8]. A scan of Middle Eastern countries such as Qatar, Kuwait, and United Arab Emirates tobacco control policies did not find any evidence of plain packaging implementation in the region. However, one country with notable forward-looking momentum is Syria. Syria has announced plans to implement plain packaging laws in 2027, which highlights a regional impetus toward adoption [8]. The lack of such plans in other Middle Eastern counties poses a clear gap in tobacco control measures. To provide a regional overview, Table 2 summarizes adult smoking prevalence alongside the quality of tobacco packaging regulations and the percentage of pack coverage across selected Middle Eastern countries. The data highlight considerable variation in both prevalence rates and regulatory approaches, ranging from text-only warnings in Lebanon, Palestine, and Syria to more comprehensive plain packaging policies with large graphic warnings in Saudi Arabia, Oman, and Turkey. This comparison underscores the uneven implementation of WHO FCTC Article 11 measures in the Middle East and illustrates the need for stronger, standardized packaging regulations to reduce tobacco use and enhance public health impact.

### 3.3. Cost-Free Implementation

Plain packaging is widely recognized as a cost-effective tobacco control measure, as the financial responsibility for redesigning packages falls primarily on the tobacco industry, while governments incur only minimal expenses related to implementation and enforcement. Evidence from the WHO Framework Convention on Tobacco Control confirms that industry actors are tasked with adapting packaging designs, whereas public sector costs are largely limited to regulatory oversight and compliance monitoring [17]. Although early adopters such as Australia faced potential litigation, the global landscape has shifted considerably: since its introduction, at least 42 countries have implemented plain packaging [52], and legal challenges from the tobacco industry have largely subsided as the policy has become normalized outside the Middle East. This trajectory suggests that the reduced likelihood of litigation should serve as a catalyst for broader adoption of plain packaging across the Middle Eastern region.

### 3.4. Starting Point Towards Tobacco Endgame

Plain packaging can signal the beginning of more efforts towards a tobacco endgame strategy. The overarching endgame goal of achieving a target usage level of under 5% of the population using tobacco products by a set date is increasingly becoming more common, and many countries that commit to this goal utilize plain packaging as part of their endgame strategies [14]. Plain packaging also seems to occur alongside other aggressive measures against tobacco use [53]. The public mindset in some instances shows great support for a world where selling cigarettes is phased out [54]. Plain packaging, relative to other measures (such as an age-based tobacco-free generations, where people born after a certain year are banned from ever using tobacco) is likely less burdensome for countries with no tobacco endgame measures, making it an ideal starter policy choice. Plain packaging is primarily a manufacturer-level regulation, limiting the burden placed on enforcement services, retailers, and governing bodies. Whereas tobacco-free generation policies for example require continuous and frequent enforcement, significant retailer compliance monitoring, and identity-checking systems all of which take substantial time and resources to implement. The implementation of plain packaging could encourage the consideration of other tobacco endgame policies in Middle Eastern countries.

### 3.5. Support for Plain Packaging Policies

Embracing policy change is largely related to public opinion and support. There is clear support for standardized packaging in the Middle East—for instance, in Qatar a study that examined how participant sociodemographic variables can influence their support for tobacco endgame policies found that for males and current tobacco users their preferred tobacco policy is standardized packaging. This suggests that their preferred policies involve messaging approaches instead of tobacco use interventions [55]. Similarly, following implementation in Saudi Arabia, the opinions on the policy and its impact are favorable to public health [56]. This support is consistent with global public support for tobacco endgame policies, including plain packaging [57]. A summary of 47 studies that were conducted throughout Australia, New Zealand, the United States, Europe, South America, Asia, and Oceania found that the majority of the general population supports tobacco policies such as limiting nicotine, banning cigarette sales, and increased taxes on tobacco products [57]. Importantly, polices are more positively perceived after their enactment through legislation, and even more so if the policy itself supports efforts to quit tobacco use [57].

### 3.6. Emerging Products Are Posing Issues

Finally, the sixth reason to support plain packaging is that new and emerging products, which are often less controlled than existing nicotine and tobacco products, pose a risk to populations, particularly youth, in becoming addicted to nicotine. Nicotine-based e-cigarette use is prevalent in the Middle East [58], and although studies about the prevalence of smokeless tobacco (such as snus) are rare, there is some evidence to suggest the degree of risk is significant. For example, a recent study found a high prevalence of 20% use of smokeless tobacco in the general population in Riyadh, Saudi Arabia [59]. As the nicotine industry expands the development of new tobacco and nicotine delivery systems, use of these emerging products will continue to increase. Products such as oral nicotine pouches are gaining widespread popularity [59]; manufactures of modern products like nicotine pouches design their products with sleek, colorful, candy like container designs which are meant to grab the attention of youth customers. Thus highlighting that the time to implement plain packaging laws is now. Plain packaging on all nicotine-based products, whether they contain tobacco or not, is a key factor in increasing awareness and deterring the appeal of such products.

Together, these six arguments, plain packaging’s proven effectiveness, its current underutilization in the Middle East, cost-free implementation, contribution to advancing tobacco endgame strategies, strong policy support, and the urgent need to address emerging nicotine products, provide a comprehensive rationale for adopting plain packaging across the region. A consolidated overview of these key points is presented in Table 3, which summarizes the case for plain packaging in the Middle East.

## 4. Discussion: Implementation Challenges, Barriers, and Mitigation Strategies

Despite the growing global momentum behind plain packaging policies, implementation in the Middle East remains constrained by a complex mix of political, cultural, economic, and industry-related barriers. Unlike high-income Western contexts, these challenges are more strongly shaped by prevailing social norms and perceptions surrounding tobacco use, compounded by well-documented interference from the tobacco industry.

From a political standpoint, tobacco control policies, including plain packaging, may be delayed or weakened due to competing government priorities, insufficient enforcement capabilities, and the necessity of balancing public health goals with economic development initiatives. In several Middle Eastern countries, where tobacco taxation and regulation are still developing, there may be limited policy coherence across ministries (e.g., health, trade, finance, and commerce), which could slow legislative adoption or weaken enforcement. Evidence from Saudi Arabia shows that the adoption of plain packaging was met with significant resistance from the tobacco industry [15]. This resistance included challenges related to policy justification and regulatory acceptance, as well as the involvement of multiple government entities responsible for health, commerce, and enforcement. These challenges highlight the complexity of policy coordination and the potential for stakeholder contestation, which can impede the implementation of effective tobacco control measures. This issue reflects broader challenges in aligning public health objectives with economic and regulatory considerations.

Culturally, tobacco use is embedded within social norms in parts of the region, especially with regard to waterpipe (shisha) use and patterns of tobacco consumption related to gender. The social acceptance of waterpipe use often exceeds that of cigarette smoking, which can vary in terms of stigma. This normalization can diminish the perceived urgency of plain packaging policies and potentially erode public support, especially when the health risks are not fully recognized. The social acceptance of waterpipe use in Middle Eastern populations is evident, with its widespread practice observed in various settings, including cafés and social gatherings, as well as family gatherings [60]. People often perceive it as being less harmful than cigarette smoking, and cultural identity, peer influence, and leisure-oriented social norms influence it [60]. This may lead to a decrease in the perceived necessity for tobacco control measures, such as plain packaging, and it can diminish public support when health risks are not taken seriously.

The implementation experience in Saudi Arabia highlights both the challenges and the mitigation strategies associated with plain packaging. As a regional leader in tobacco control, Saudi Arabia has advanced packaging and labelling regulations within a broader national strategy aligned with WHO Framework Convention on Tobacco Control (FCTC) commitments. However, this process has required sustained regulatory enforcement and cross-sectoral coordination to counter industry resistance and ensure compliance. Public awareness initiatives have also been critical to foster understanding and acceptance of standardized packaging, particularly in a context where social norms around tobacco use, such as waterpipe smoking, remain influential.

To overcome these barriers, several practical strategies are recommended. First, continuous communication campaigns are needed to raise awareness of tobacco harms and build support for plain packaging, especially among youth and in relation to waterpipe use. Second, stakeholder engagement should involve coordinated action across ministries of health, education, finance, and trade, together with civil society organizations, to strengthen policy coherence and implementation capacity. Third, legal preparedness is essential, requiring robust legislation aligned with international trade and intellectual property frameworks to anticipate and defend against industry litigation. Finally, systematic monitoring of tobacco industry interference should be institutionalized, including transparency mechanisms for lobbying activities and adherence to Article 5.3 of the WHO FCTC, to safeguard policy development from commercial influence.

Collectively, these measures can improve the feasibility and sustainability of plain packaging in the Middle East, reinforcing its role as a comprehensive tobacco control strategy.

### 4.1. Future Directions

As we have outlined above, there remains a significant amount of work to be done in Middle Eastern countries to better control tobacco and nicotine product marketing, and to move toward a tobacco endgame strategy. First, there is a dearth of research in the Middle East on the impact of plain packaging on people’s perceptions of, purchasing, and use of tobacco and nicotine products [30]. It would therefore be useful to get a baseline assessment of attitudes toward plain packaging in Middle Eastern countries where plain packaging has not yet been implemented. Additionally, future research should also focus on contributing to key policy implication areas. This would include looking at implementation strategies, evaluation metrics, resources and commitment to policy change, consequences of manufacturer burden, and better understanding the socio-cultural impact of implementation.

When plain packaging was introduced in Saudi Arabia, smokers quickly accused the government and the tobacco companies of trying to sell an inferior tobacco product [15]. Therefore, future research should include investigations of attitudes toward tobacco products, government, and the tobacco companies when introducing plain packaging. As noted previously, Syria is scheduled to implement plain packaging in 2027 [8], this will create a natural experiment. Providing a unique opportunity for a longitudinal pre-post study to understand how implementation affects public attitudes and consumption.

Another area of interest would be to conduct research on public perceptions of new nicotine products (such as vapes, snus, flavored tobacco, etc.). This could be particularly relevant in the region due to the more permissive smoking culture of the Middle East, particularly for males [18]. Although cigarette and waterpipe use are the most common type of tobacco product used, the increased prevalence of vaping and other alternative nicotine delivery products may circumvent cultural, social, and health pressures to reduce use of tobacco and nicotine products.

### 4.2. Limitations

It is imperative to acknowledge the several limitations of this review. First, there is the potential for selection bias in this narrative review. That is because the studies included were chosen to illustrate key themes, not because they were selected through a systematic process. Second, the ability to establish causality is limited by the design of narrative reviews, as definitive cause-and-effect relationships cannot be interpreted from the associations between plain packaging and tobacco outcomes. Third, variations in study quality and contexts are reflected in the evidence base, with methodological differences, sample population differences, and cultural setting differences that may affect generalizability across the Middle East. Fourth, although plain packaging is often described as low-cost for governments, costs remain for implementation and enforcement, particularly for monitoring compliance and addressing concerns about illegal trade, these costs are hidden and difficult to estimate without transparent data. Finally, alternative nicotine products are a concern for implementation as they have diverse shapes which poses challenges in applying a blanket conclusion on the effectiveness of this measure without actual studies of each product. Further, these products may not yet be governed by the same packaging regulations. The effectiveness of plain packaging policies could be undermined as a result.

## 5. Conclusions

Plain packaging represents a practical, cost-effective, and evidence-based tobacco control intervention that aligns with broader tobacco endgame strategies. The added value of this manuscript lies in three elements: (a) its dedicated focus on the Middle East, where tobacco use is shaped by social norms, waterpipe smoking, and emerging nicotine products; (b) the application of the Ottawa Charter framework to systematically map multisectoral health impacts; and (c) the development of a structured “six reasons” policy argument that consolidates public health, behavioural, economic, and implementation rationales.

From the perspective of the Ottawa Charter, plain packaging advances healthy public policy by restricting industry branding, creates supportive environments by reducing product appeal and reinforcing health warnings, strengthens community action through collective advocacy, develops personal skills by enhancing risk awareness, and reorients health services toward prevention.

Positioned within the broader global literature, this review distinguishes itself from existing systematic reviews that primarily emphasize behavioural outcomes in early-adopting high-income countries such as Australia, the UK, France, Canada, and New Zealand. By centering the Middle East and applying a health promotion framework, it extends the evidence base beyond effectiveness to provide a context-sensitive policy interpretation.

Overall, the effectiveness of plain packaging, its low implementation costs, and growing international adoption underscore the urgency of expanding this measure to both combustible tobacco and emerging nicotine products in the Middle East. Ultimately, plain packaging reduces product appeal, supports informed decision-making, and advances long-term public health objectives in line with global tobacco control commitments.

## Figures and Tables

**Table 1:** Examples of key studies on plain packaging, including country context, study design, and main findings.

Study	Examples of Key Studies on Plain Packaging	Country/Context	Design	Main Findings	Source
Hassounah et al., 2020	Implementation of Cigarette Plain Packaging: Triadic Reactions of Consumers, State Officials, and Tobacco Companies—The Case of Saudi Arabia	Saudi Arabia/Following implementation of plain packaging for cigarettes in August 2019, a social media campaign (#الدخان_الجديد “the new smoke”) emerged, with smokers claiming that cigarettes tasted different after the packaging change.	Qualitative case study. Researchers constructed a timeline of events and conducted online social listening of Arabic Twitter hashtags related to the campaign. They analyzed reactions from consumers, government agencies, media, health advocates, and tobacco companies.	Consumer complaints: ○ Campaigners primarily criticized plain packaging, claiming it caused an unfavorable new taste in cigarettes. Escalation of messaging: ○ Complaints evolved into accusations directed at government agencies and neighboring countries, followed by threats to boycott tobacco companies. Media and official response: ○ The campaign attracted significant media coverage. ○ Saudi government bodies, including the Saudi Food and Drug Authority and the Ministry of Commerce and Investment, issued official responses.	[15]
Mansour et al., 2017	Factors Affecting the Perceived Effectiveness of Pictorial Health Warnings on Cigarette Packages in Gulf Countries: A Cross-sectional Study	Gulf Cooperation Council (GCC) countries. The study assessed the effectiveness of the GCC-required pictorial health warnings (PHWs) on cigarette packs and compared them with US FDA-approved warning labels.	Cross-sectional study using a convenience sample of adult smokers, former smokers, and non-smokers. Data were collected through an online self-administered questionnaire. Participants rated the perceived effectiveness of different warning labels.	Survey participation: ○ Of 90 invited individuals, 77 completed the survey (86% response rate). ○ Composition: 39 nonsmokers (50%), 22 smokers (29%), and 16 former smokers (21%). Perceived effectiveness of labels: ○ Labels with graphic images of illness or pathology were viewed as the most effective overall. ○ Smokers rated these labels significantly less effective compared with nonsmokers and former smokers. Quit-line support: ○ 55 respondents (71%) indicated that including a telephone quit-line would be an effective measure.	[41]
Abu-Rmeileh et al., 2022	Tobacco control in the Eastern Mediterranean region: implementation progress and persisting challenge	Eastern Mediterranean Region (EMR), covering implementation of WHO FCTC measures across 22 countries. Includes discussion of tobacco packaging and labeling policies	Regional policy review/commentary based on analysis of FCTC implementation indicators, WHO reports, and regional tobacco control policies.	Mandated warnings: ○ All Eastern Mediterranean Region (EMR) countries, except Somalia, require health warnings on cigarette packs. ○ The EMR ranks second globally after Southeast Asia, where all countries mandate warnings. Pictorial health warnings (PHWs): ○ Several EMR countries have implemented PHWs, but coverage size varies. ○ Examples: Pakistan requires 60% of the pack, while Jordan mandates 40%. ○ Lebanon requires textual and pictorial warnings to cover 40% of tobacco packs, including waterpipe products. Egypt mandates 50% coverage. Plain packaging adoption: ○ In 2019, Saudi Arabia became the first EMR country—and among the first worldwide—to introduce plain packaging.	[42]
Awaisu et al., 2013	Pictorial Health Warnings on Cigarette Packages in Qatar: Preimplementation Awareness and Perceptions of Ever-Smokers Versus Never-Smokers	Qatar. Conducted shortly before implementation of pictorial health warnings (PHWs) on cigarette packs in Qatar. The study assessed public awareness and perceptions of tobacco warning labels among ever-smokers and never-smokers in Doha.	Cross-sectional survey using a pretested 23-item questionnaire administered via face-to-face interviews. A total of 500 adults were recruited from public places. Participants were categorized as ever-smokers (current or former smokers) and never-smokers. Statistical comparisons were made using chi-square/Fisher’s exact tests.	Awareness of PHWs: ○ Ever-smokers and never-smokers showed no significant difference in awareness of pictorial health warnings (PHWs). ○ About one-third of respondents were unaware of any specific text warnings. ○ Nearly 45% did not know what a PHW was. Perceived effectiveness: ○ Over 20% of respondents in both groups did not believe PHWs would influence smoking behavior change. Attitudinal differences: ○ Nonsmokers generally expressed more positive attitudes toward the potential impact of PHWs compared with smokers.	[43]
Jradi et al., 2018	Graphic warnings and text warning labels on cigarette packages in Riyadh Kingdom of Saudi Arabia Awareness and perceptions	Riyadh, Saudi Arabia. The study evaluated public awareness and perceptions of graphic health warnings and text warning labels on cigarette packages following implementation of GCC-standard tobacco packaging regulations (implemented in 2012).	Cross-sectional qualitative study using nine focus group discussions (health-care workers, adult men, adult women) and semi-structured interviews with community leaders (including religious leaders). Participants were shown actual cigarette warning labels used in Saudi Arabia and asked about awareness and perceptions.	General awareness and support: ○ Most participants were aware of graphic warning labels (GWLs) on cigarette packs and supported their use. Detail recognition: ○ Awareness of the specific content and details on the labels was relatively low. Perceived effectiveness: ○ Participants viewed current labels as somewhat vague in promoting cessation and called for stronger, more aggressive graphics. Community leader perspectives: ○ Leaders favored text-only warnings and opposed aggressive graphic labels, considering them culturally and religiously inappropriate.	[44]
Alshahrani et al., 2020	Need for Tobacco Plain Packaging in the Kingdom of Saudi Arabia: A Groundbreaking Step for Smoking Control Measure.	Saudi Arabia. The paper discusses the potential role of plain tobacco packaging as part of tobacco control policy in Saudi Arabia, in the context of rising tobacco use and existing FCTC-aligned regulations (including pictorial warnings and advertising restrictions).	Policy commentary/narrative viewpoint article. The author draws on existing global evidence on plain packaging (particularly Australia and other early adopters) and applies it to the Saudi context.	Industry marketing strategy: ○ Tobacco companies have relied heavily on packaging as a primary advertising tool to influence youth smoking behavior. Policy adoption: ○ Australia pioneered standardized plain packaging in December 2012, later endorsed by the WHO as a global public health measure. ○ Saudi Arabia adopted plain packaging in May 2019, following WHO recommendations, despite legal challenges and consumer resistance. Consumer response: ○ Tobacco companies opposed the policy, and some consumers launched campaigns claiming taste and quality changes in cigarettes. Evidence of effectiveness: ○ Quantitative and qualitative studies show plain packaging reduces positive perceptions of smoking and encourages cessation. Policy coordination: ○ Effective implementation requires collaboration among the Ministry of Health, Saudi Food and Drug Authority, and Ministry of Commerce. ○ Social media and web-based communication can help address consumer concerns and raise awareness. Compliance and standards: ○ Ensuring tobacco companies adhere to international manufacturing standards is critical for sustaining the policy.	[45]
Mostafa et al., 2016	Tobacco packaging and labelling policies in countries of the Eastern Mediterranean and Western Pacific Regions: Post-deadline assessment of the time-bound measures of WHO FCTC. (WHO Report/Policy evaluation).	Countries in the Eastern Mediterranean Region (EMR) and Western Pacific Region (WPR). The report evaluates national compliance with WHO Framework Convention on Tobacco Control (FCTC) Article 11 time-bound obligations on packaging and labelling (including health warnings and plain packaging progress where applicable).	Cross-sectional policy compliance assessment using WHO FCTC implementation reports, Party submissions, and regional monitoring data after the deadline for implementing Article 11 packaging and labelling requirements.	Adoption of text warnings: ○ 44 countries (90%) initially adopted text-based health warnings. Shift to pictorial warnings (PHWs): ○ After ratifying the Convention, 25 countries (51%) introduced PHWs. ○ Regional breakdown: 13 countries (59%) in the Eastern Mediterranean Region (EMR) and 12 countries (44%) in the Western Pacific Region (WPR). Compliance with deadlines: ○ Only 11 countries (44%) met the required implementation deadline. ○ Overall, just 10 countries (20%) achieved high compliance with time-bound measures, and none fully complied with all 15 requirements. Most and least common measures: ○ The most widely adopted measure was mandating health warnings in national tobacco control laws (90%). ○ The least adopted was banning flavor descriptors, implemented by only 4% of countries.	[46]
Bader et al., 2018	Informing tobacco control policy in Jordan: assessing the effectiveness of pictorial warning labels on cigarette packs. (Jordan policy evaluation study).	Jordan. The study evaluated the introduction of pictorial health warning labels (PHWs) on cigarette packs as part of Jordan’s implementation of WHO FCTC Article 11 packaging and labeling requirements.	Cross-sectional mixed-methods study using survey data and perception assessment of smokers and non-smokers exposed to cigarette packaging with pictorial warnings. Participants were assessed on awareness, recall, and perceived effectiveness of warning labels.	Awareness and recall: ○ Both smokers and non-smokers were aware of the pictorial warning label (PWL) set and could recall at least one message. Effectiveness of warnings: ○ One-third of smokers who frequently viewed the warnings reported that PWLs prompted them to consider quitting. ○ In 2015, the set continued to motivate both smokers and non-smokers to quit or remain smoke-free. Decline in salience: ○ Evidence suggested the warnings’ impact had diminished over time, indicating the set had reached the end of its effective lifespan. Variability among warnings: ○ Performance differed across individual PWLs. ○ The warning depicting human suffering was significantly more effective, with its motivational impact strongly linked to evoking fear.	[47]
Caner et al., 2023	Perceptions of plain packaging and health warnings among university students in Turkey: a survey-based experiment.	Turkey (Ankara). University students (ever-smokers) were exposed to experimental cigarette pack images varying by packaging type (branded vs. plain) and health warning strength (old vs. new graphic warnings). The study was conducted in 2021 among students from four universities in Ankara.	Online survey-based randomized experiment (n = 623 ever-smokers). Participants were randomly assigned to one of five conditions showing different combinations of branded/plain packaging and old/new graphic health warnings. Outcomes measured: negative affect, avoidant responses, and intention to quit within 6 months using regression analysis.	Impact of stronger graphic health warnings (GHWs): ○ Produced greater negative emotional responses (+0.31/5, *p* = 0.010). ○ Increased avoidant reactions (+0.42/5, *p* = 0.002) compared with older warnings where brand logos were visible. Impact of plain packaging (PP): ○ Generated stronger negative affect (+0.48/5, *p* < 0.001). ○ Increased avoidant responses (+0.46/5, *p* = 0.001) compared with branded packs carrying old warnings. Combined effects: ○ When disentangling PP and new GHWs, neither independently influenced smokers’ intentions to quit within six months.	[10]
Al-Ahmadi et al., 2024	Perceptions of health warnings on cigarette sticks among the adult population in Al-Madinah, Saudi Arabia: A cross-sectional survey	Al-Madinah, Saudi Arabia. The study assessed adult perceptions of health warnings on cigarette sticks (novel dissuasive cigarettes) and compared them with perceptions of standard cigarette packaging warnings. It also evaluated acceptance of implementing stick-based warnings as a tobacco control measure.	Cross-sectional survey (n = 285 adults, smokers and non-smokers). Data collected via online questionnaire (WhatsApp distribution). Participants were exposed to images of cigarette pack warnings and cigarette stick warning messages (mortality, health consequences, social/financial consequences, supportive quit messages). Analysis included chi-square tests and logistic regression.	Effectiveness of package warnings: ○ 18.6% of participants rated package warnings as “quite effective” or “very effective” in encouraging smokers to quit. Health warnings on cigarette sticks: ○ 28.1% perceived mortality-related statistics as effective in prompting quitting. ○ 35.0% found themes highlighting social and financial consequences more effective. Influence of education level: ○ Respondents with secondary education or lower were nearly twice as likely to support health warnings compared with those holding university degrees or higher (OR = 1.9; 95% CI: 1.02–3.7, *p* = 0.042). Overall sentiment: ○ Most participant comments expressed positive support for including health warnings on cigarette sticks.	[48]
Aljuaid et al., 2020	Taxation and tobacco plain packaging effect on Saudi smokers quitting intentions in Riyadh city, Saudi Arabia.	Riyadh, Saudi Arabia. The study examined the effect of tobacco taxation and plain cigarette packaging on smokers’ quitting intentions among adult male smokers. Participants were recruited from public smoking areas (coffee shops) across Riyadh.	Cross-sectional quantitative survey (n = 1015 male smokers; final analyzed sample n = 960). Data were collected using a structured questionnaire including exposure to plain packaging images and questions on taxation perceptions and quitting intention. Logistic regression and chi-square tests were used for analysis.	Taxation impact: ○ 46.5% of participants reported they would not quit smoking if cigarette prices increased. ○ Among those already planning to quit, higher prices significantly strengthened their intention to stop (*p* < 0.001). Plain packaging perceptions: ○ Logistic regression identified predictors of quitting intention. ○ Interestingly, participants opposed to applying plain packaging in Saudi Arabia were more likely to express quitting intentions compared with those supportive of the policy (Odds Ratio: 2.30; 95% CI: 1.61–3.28).	[21]

**Table 2:** Adult smoking prevalence and quality of tobacco packaging regulation in Middle Eastern countries.

Country	Adult Smoking Prevalence (%)	Quality of Tobacco Packaging Regulation	% of Pack Covered	Source
Bahrain	18.50% [50]	Text warning label with graphic warning label [51]	50% [51]	[50,51]
Egypt	24.90% [50]	Text warning label with graphic warning label [51]	50% [51]	[50,51]
Iran	8.40% [50]	Text warning label with graphic warning label [51]	50% [51]	[50,51]
Iraq	19.30% [50]	Text warning label with graphic warning label [51]	50% [51]	[50,51]
Israel	20.50% [50]	Plain packaging without graphic warning labels [51]	65% [51]	[50,51]
Jordan	34.70% [50]	Text warning label with graphic warning label [51]	40% [51]	[50,51]
Kuwait	24.50% [50]	Text warning label with graphic warning label [51]	50% [51]	[50,51]
Lebanon	47.00% [50]	Text warning label only [51]	40% [51]	[50,51]
Oman	10.70% [50]	Plain Packaging with text/graphic warning label [51]	65% [51]	[50,51]
Palestine	NA	Text warning label only [51]	10% [51]	[51]
Qatar	17.40% [50]	Text warning label with graphic warning label [51]	50% [51]	[50,51]
Saudi Arabia	16.60% [50]	Plain Packaging with text/graphic warning label [51]	65% [51]	[50,51]
Syria	NA	Text warning label only [51]	15% [51]	[51]
United Arab Emirates	11.10% [50]	Text warning label with graphic warning label [51]	50% [51]	[50,51]
Yemen	6.70% [51]	Text warning label with graphic warning label [51]	50% [51]	[51]
Turkey	30.80% [50]	Plain Packaging with text/graphic warning label [51]	92.5% [51]	[50,51]

**Table 3:** The Case for Plain Packaging in the Middle East.

Key Reasons	Justification
Effectiveness	Evidence from multiple countries and substances demonstrates that plain packaging decreases product attraction, heightens warning recognition, and increases negative product perceptions which contributes to reduced consumption and initiation.
Underutilization	Plain packaging remains limited in the Middle East. Regional differences in regulation and an uptake in nicotine product users reveals an opportunistic time to implement plain packaging.
Cost-free Implementation	Plain packaging is a cost-effective tobacco control policy as implementation and enforcement costs are low. The cost burden is limited to the tobacco industry at the manufacturing level.
Tobacco Endgame	Plain packaging can act as a low risk, high reward foundational tobacco endgame policy. As a low-burden intervention, it may facilitate the adoption of more comprehensive control measures and build momentum for additional endgame policy changes.
Policy Support	The evidence from public opinion research indicates support for tobacco control policies in the Middle East and other regions, with acceptance often increasing after implementation.
Emerging Products	With a rise in emerging nicotine products, plain packaging offers a proactive approach to address growing public health concerns by reducing product appeal and increasing risk awareness.

## Data Availability

No new data were created or analyzed in this study.

## Abbreviations

The following abbreviations are used in this manuscript:

WHO	World Health Organization
FCTC	Framework Convention on Tobacco Control
